# The effectiveness of the median nerve neurodynamic mobilisation techniques in women with mild or moderate bilateral carpal tunnel syndrome: A single-blind clinical randomised trial

**DOI:** 10.4102/sajp.v78i1.1823

**Published:** 2022-11-30

**Authors:** Hassan Beddaa, Bouchra Kably, Basma Marzouk, Ikrame Mouhi, Abdelghafour Marfak, Youness Azemmour, Ismail Bouzekraoui Alaoui, Nazha Birouk

**Affiliations:** 1 Clinical Research Biostatistics and Epidemiology Laboratory, Faculty of Medicine and Pharmacy, Mohammed V University, Rabat, Morocco; 2 Department of Clinical Neurophysiology, Specialty Hospital, Ibn Sina University Hospital Center, Mohammed V University, Rabat, Morocco; 3 National School of Public Health, Rabat, Morocco; 4 Moroccan National Olympic Committee, Rabat, Morocco

**Keywords:** neurodynamic mobilisation techniques, carpal tunnel syndrome, manual therapy, median nerve, grip strength, pain, functional status

## Abstract

**Background:**

Carpal tunnel syndrome (CTS) is the most prevalent upper limb compression neuropathy. Surgical or nonsurgical treatment is recommended. Both mild and moderate CTS can be managed conservatively. Neurodynamic mobilisation techniques (NMTs) of the median nerve have not been widely studied, and conflicting findings exist.

**Methods/design:**

Sixty-two female patients with mild or moderate bilateral CTS were assigned one wrist to the treatment group (TG) and the other to the control group (CG). Both groups underwent carpal bone mobilisation. The TG underwent NMTs while the CG received a placebo elbow mobilisation not targeting the median nerve. The Numerical Rating Pain Scale, JAMAR Plus Digital Hand dynamometer and Functional Status Scale (FSS) were used to assess pain, grip strength and functional status.

**Discussion:**

Comparison of groups showed that NMTs at 5 weeks decreased pain intensity by 1.15 (*p* = 0.001) and by 2 (*p* ˂ 0.001) at 10 weeks. Difference in functional status was 0.45 at 5 weeks (*p* = 0.003) and 0.84 at 10 weeks (*p* = 0.003). The CG’s grip strength improved by 0.59 (*p* = 0.05) after 5 weeks and 0.61 (*p* = 0.028) at 10 weeks. Both groups improved in all parameters over time.

**Conclusion:**

When combined with carpal bone mobilisation, both NMTs and placebo elbow mobilisation seem to reduce pain intensity and improve grip strength and functional status. However, NMTs had better results in pain intensity and FSS.

**Clinical implications:**

Women with mild or moderate bilateral CTS may benefit from NMTs as a conservative treatment option.

**Trial registration:**

Pan African Clinical Trials Registry, PACTR202201807752672, https://pactr.samrc.ac.za/TrialDisplay.aspx?TrialID=19340.

## Introduction

Carpal tunnel syndrome (CTS) is the upper limb’s most commonly occurring compression neuropathy (Aroori & Spence 2008). It is a result of the compression of the median nerve under the transverse carpal ligament in the carpal tunnel (Chammas et al. 2014). Its prevalence varies in different populations, and it is more frequent among women and the working class (Dale et al. 2013; Kozak et al. 2015; Papanicolaou, McCabe & Firrell 2001; Tuppin et al. 2011; You, Smith & Rempel 2014). Bilateral presentation of CTS is quite common (Bagatur & Zorer 2001; Dec & Zyluk 2018). The diagnosis of this syndrome is based on the history, clinical examination, ultrasound and nerve conduction studies (NCS) (Jablecki et al. 2002; Koyuncuoglu et al. 2005).

Pain, loss of handgrip strength, numbness and tingling over the median nerve distribution are the most prominent signs of this condition (Leblanc & Cestia 2011). Furthermore, absenteeism from work, limited activity and significant discomfort are the results of this condition (Fernández-De-Las-Peñas et al. 2015a; Katz et al. 1998; Saint-Lary et al. 2015).

The early management of CTS may limit the damage to the median nerve and therefore improve patients’ quality of life and reduce the management costs of this neuropathy (Brininger et al. 2007; Hobson-Webb & Juel 2017). Because of this, surgical and nonsurgical treatments are recommended as part of CTS management. Compared to conservative treatment, surgery has better outcomes (Shi & MacDermid 2011). However, carpal tunnel release’s cost and side effects are such that a more conservative procedure is usually sought (Fernández-De-Las Peñas et al. 2015b). Several methods are employed in conservative treatments for CTS like splints, functional massage, carpal bone mobilisation, ultrasound therapy, electrophysical modalities, kinesiotaping, tendon gliding and neurodynamic mobilisation techniques (NMTs) of the median nerve (Ballestero-Pérez et al. 2017; Boudier-Revéret et al. 2017; Kim 2015; Pinar & Ada 2005; Schmid et al. 2012; Sim et al. 2019; Wolny et al. 2017).

Neurodynamic mobilisation techniques are a part of manual therapy that focus on diagnosing and treating specific disorders, including the peripheral nervous system. These techniques are widely used in CTS management (Wolny 2017). A recent clinical trial showed the long-term effectiveness of NMTs in improving pain intensity, grip strength and functional status (Hamzeh et al. 2021). Wolny et al. (2017) showed that manual therapy, including neurodynamic techniques, positively affected pain reduction and functional status in people with CTS (Wolny et al. 2017). Some recent systematic reviews, however, found that the NMT results are inconclusive, with limited evidence about their effectiveness in managing this condition (Ballestero-Pérez et al. 2017; Núñez De Arenas-Arroyo et al. 2021).

According to Wolny et al. (2017), including a placebo treatment group (TG) in their study would have helped to quantify its effect (Wolny et al. 2017). A year later, they conducted a randomised placebo-controlled trial in an intermediate position and without neurodynamic sequences in their sham therapy group. They found that NMTs have superior therapeutic effects on pain intensity and function but not on grip strength (Wolny & Linek 2018). Previously, Bialosky et al. (2009) conducted a similar trial with a sham therapy group that received repetitive passive mobilisation of the wrist and fingers into flexion and extension, alternately in an intermediate position, without stressing the median nerve. In both the NMTs and placebo groups, pain intensity and upper limb disability improved. The study’s conclusion also addressed the success of their sham intervention in blinding the participants (Bialosky et al. 2009).

Our study aimed to investigate the effectiveness of NMTs of the median nerve compared to joint mobilisation of the elbow not directed at the median nerve on pain intensity, grip strength and functional status in women with mild or moderate CTS.

## Methods

### Study design

A randomised single-blind clinical trial was conducted at the clinical neurophysiology department of the Rabat Specialty Hospital (RSH) between March 2019 and December 2020.

### Participants

Patients diagnosed with mild or moderate bilateral CTS by a neurologist and confirmed by NCS were invited to participate in our study. The recruitment was carried out in the clinical neurophysiology department of RSH. Patients had to be at least 18 years old and could read and write Arabic to participate. Exclusion criteria included age under 18 years, pregnancy, any upper limb range of motion limitation for other reasons than CTS, other peripheral neuropathies, thenar muscle atrophy, cervical radiculopathy, inflammatory joint disease in the upper limbs, any CTS treatment received within the last 3 months of enrolment, systemic disease, unilateral CTS and diabetes.

### Sample size

The patient recruitment period was previously set at 18 months from March 2019. However, this period coincided with the government-imposed 3-month confinement, which caused our study to be extended to December 2020.

The required sample size was calculated based on prior data to detect a treatment difference of 0.74 units on the Boston Carpal Tunnel Syndrome Questionnaire (BCTQ) Functional Status Scale (FSS) (Kim & Jeon 2013). Therefore, 50 participants were considered sufficient for our study. The sample size calculation was performed using Statulator (Navneet Dhand and Mehar Khatkar in Sydney, Australia), assuming a standard deviation of 1.0, a superiority margin of 0.24, an alpha significance level set at 0.05 and statistical power of 0.80. It was expected that 20% of the patients would drop out, so our study tried to get at least 60 patients.

### Diagnostic criteria for carpal tunnel syndrome

Based on the history, clinical examination and NCS, a neurologist with 20 years of experience in electrodiagnostic testing diagnosed bilateral CTS. The criteria of NCS adopted by the clinical neurophysiology department of the RSH are compatible with the American Association of Electrodiagnostic Medicine recommendations (Jablecki et al. 2002).

### Randomisation and blinding procedures

All individuals diagnosed with mild or moderate bilateral CTS who met the diagnostic criteria were eligible to participate. A random draw was made for each participant who was not excluded in determining which wrist would be assigned to the TG. Each wrist was then randomly allocated to either the TG or control group (CG). For example, those who drew ‘right’ had the right wrist assigned to the TG, and the left wrist was automatically assigned to the CG and vice versa. The random selection of the wrist to be treated with the NMTs of the median nerve was supervised by a secretary who was not involved in our study. The patient was then referred to a physiotherapist to complete the general data of the examination form, the medical-surgical history and the clinical examination to assess the level of pain intensity using the Numerical Pain Rating Scale (NPRS), grip strength with the JAMAR Plus+ Digital Hand dynamometer and the FSS of the BCTQ. The intervention was performed by another physiotherapist who was aware of the wrist to be treated with NMTs but unaware of the examination results. At 5 weeks and immediately after the treatment cycle, the first physiotherapist, who was unaware of the treated wrist, performed the second and third assessments.

### Outcome measures

A blinded physiotherapist performed all the outcome measures at baseline, after 5 weeks and after 20 sessions at 10 weeks.

Physical ability and functional status were assessed using the FSS, part of the BCTQ. This questionnaire contains another scale that addresses symptom severity and was not used here. The FSS includes eight items that need to be rated on a five-point Likert scale for difficulty level. A final score ranging from 1 to 5 is generated from this scale, where a higher score indicates a greater disability (Levine et al. 1993). The Arabic version of the BCTQ-FSS translated and validated by Alanazy was used (Alanazy et al. 2019). All participants completed a separate FSS questionnaire for each wrist at baseline, after 5 weeks and immediately after the treatment cycle at 10 weeks.

Pain intensity in the median nerve sensory area was assessed using the NPRS (0 = no pain, 10 = maximum pain) separately in each hand for all patients. A greater number corresponds to a higher level of pain and vice versa. Compared to similar tools, the NPRS showed better compliance (Hjermstad et al. 2011). Assessment of the level of pain intensity was performed at baseline, after 5 weeks and immediately after the treatment cycle at 10 weeks.

Hand grip strength was measured (in kg) using a digital hand dynamometer (JAMAR Plus+). It is considered the gold standard by which other dynamometers are evaluated (Roberts et al. 2011). The mean of three consecutive trials was accepted for each force measurement. The hand grip strength evaluation was performed at baseline, after 5 weeks and immediately after the treatment cycle at 10 weeks.

### Intervention

All patients underwent 20 physiotherapy sessions for 10 weeks at two sessions per week. Every patient received mechanical interface treatment on both wrists based on carpal bone mobilisation (horizontal flexion and progressively horizontal extension), as described by Shacklock (2005), and underwent a different intervention on each wrist. Therefore, one wrist received NMTs directed at the median nerve and was then enrolled in the TG. At the same time, the other wrist received a placebo treatment based on the joint mobilisation of the elbow that was not directed at the median nerve and was then enrolled in the CG. For the TG, the first 15 sessions were devoted to ‘two-ended sliders’ mobilisation techniques of the median nerve according to the upper limb neurodynamic test 1 (ULNT1) position described by Shacklock (2005). The median nerve was slid between the wrist and elbow by maintaining this test position, mobilising them simultaneously. We mobilised the elbow in extension for the proximal direction while mobilising the wrist in flexion, and the two previous joint positions were reversed simultaneously for the distal direction. We performed four sets of 30 repetitions separated by a 30-s rest period, as Shacklock (2005) recommended. We carefully performed median nerve ‘one-ended tensioners’ techniques based on the ULNT1 position during the last five sessions, always followed by ‘sliders techniques’ as described earlier, but this time only by mobilising the wrist from extension (distal direction) to flexion (proximal direction). When no adverse reactions were produced based on the patient’s feedback during and after the technique, four sets of 30 repetitions separated by a 30-s rest period were performed. Otherwise, ‘two-ended sliders’ were used instead.

In the CG, elbow joint mobilisation not directed at the median nerve was performed as a placebo treatment. The patient was positioned in a supine position without a pillow. The cervical spine was placed in a neutral position, the shoulder in 45° abduction and external rotation, the forearm in pronation and the wrist and fingers in permanent flexion. This mobilisation consisted of mobilising the elbow from flexion to extension and immediately reversing it from extension to flexion in the midrange of motion. We carried out four sets of 30 repetitions separated by 30-s interset intervals. Bialosky et al. (2009) concluded that their study successfully blinded the participants with the sham intervention (Bialosky et al. 2009). The same method was previously used on healthy people and showed that it could be useful in clinical trials. The only difference is that we mobilised the elbow instead of the wrist, as done by Bialosky et al. and Wolny et al., to minimise the stress on the median nerve in the carpal tunnel.

### Statistical analysis

The data were recorded in an electronic database, ensuring the anonymity and confidentiality of the participants. The sample characteristics were summarised by means, standard deviations, ranges and proportions.

Adherence to the normal distribution of variables was assessed using the Kolmogorov–Smirnov and Shapiro–Wilk tests. For multiple comparisons, a one-way analysis of variance (ANOVA) was used in the case of normally distributed variables. In contrast, the Friedman test was used for the same purpose in the case of non-normally distributed variables. For the paired comparisons, the paired *t*-test was used in the case of normal data distribution; otherwise, the Wilcoxon test was performed instead. All statistical test analyses were carried out at a significant level of *p* ≤ 0.05 using the Statistical Package for the Social Sciences (SPSS) version 20 software (IBM Corporation, Armonk, New York, United States of America).

### Ethical considerations

The study was authorised by the Biomedical Research Ethics Committee at the Faculty of Medicine and Pharmacy of Rabat, part of Mohammed V University. All study procedures were performed according to the Helsinki Declaration of Human Rights of 1975 (modified in 1983). The clinical trial registration number is PACTR202201807752672.

## Results

**FIGURE 1:**
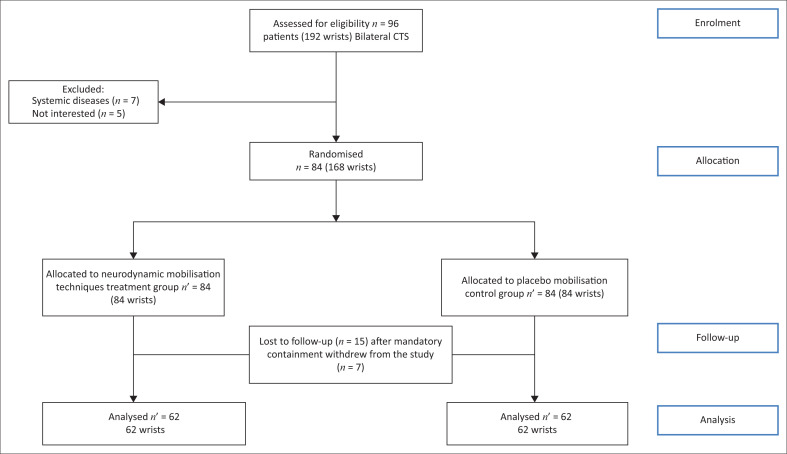
Consolidated Standards of Reporting Trials (CONSORT) flow diagram of the study.

Ninety-six participants met the inclusion criteria, of whom 12 were excluded either because of systemic disease (7 patients) or because they were not interested in our study (5 patients). In the end, 84 patients were included. The patient recruitment period coincided with the mandatory confinement imposed by the government to stop the spread of coronavirus disease 2019 (COVID-19). As a result, 15 patients were lost to follow-up after the mandatory confinement was lifted. In addition, seven patients voluntarily withdrew without giving a specific reason. In the end, 62 women (124 wrists) completed our study. Patients were randomly assigned one wrist to the TG and the other automatically to the CG. A Consolidated Standards of Reporting Trials (CONSORT) flow diagram illustrates this process (Figure 1).

All participants in our study were female. The mean age of the participants was 52.45 years (standard deviation [SD] 10.55), of whom 36 (58.1%) were housewives, and 26 were gainfully employed, of whom 12 (19.4%) were blue-collar. Their body mass index (BMI) mean was 27.30 kg/m² (SD 3.82). The random draw allocated 31 (50%) right wrists equal to left wrists to the TG (Table 1).

**TABLE 1:** Characteristics of the sample.

Characteristics of participants	*n*	%	Overall (*n* = 62)		
			Mean	SD	Minimum–maximum
Age (years)	-	-	52.45	10.55	30–78
Body mass (kg)	-	-	70.45	10.81	51–93
Height mean (m)	-	-	1.60	0.60	1.49–1.78
BMI (kg/m²)	-	-	27.30	3.82	18.92–36.33
**Number of treated side**					
Right	31	50	-	-	-
Left	31	50	-	-	-
**Number of occupation**					
Housewives	36	58.1	-	-	-
Blue-collar	12	19.4	-	-	-
White-collar	14	22.6	-	-	-

SD, standard deviation; BMI, body mass index.

The Friedman test performed on the level of pain intensity showed a positive effect of the NMTs. A mean decrease of 2.17 in pain intensity was observed after only 10 sessions and 3.65 at the end of the treatment cycle (*p* ˂ 0.001). In the CG, a decrease in pain was also observed, with a difference of 1.31 between the baseline and the end of treatment (*p* ˂ 0.001) (Table 2).

**TABLE 2:** Group means and standard deviation for pain intensity, grip strength and functional status at different time intervals in each group.

Variable	Time of observation	Treatment group			Control group		
		Mean	± SD	*p*	Mean	± SD	*p*
Pain intensity (NRPS)	Baseline	5.17	± 1.71	-	4.83	± 1.95	-
	5 weeks	3	± 1.76	-	4.15	± 1.87	-
	10 weeks	1.52	± 1.50	-	3.52	± 1.76	-
	-	-	-	**0.0001** †	-	-	**0.0001** †
Grip strength (kg)	Baseline	20.29	± 3.71	-	19.59	± 3.08	-
	5 weeks	20.50	± 3.71	-	19.91	± 3.40	-
	10 weeks	20.84	± 3.56	-	20.23	± 3.29	-
	-	-	-	**0.001** ‡	-	-	**0.012** ‡
BCTQ-FSS	Baseline	2.77	± 0.88	-	2.62	± 0.88	-
	5 weeks	2.05	± 0.76	-	2.50	± 0.86	-
	10 weeks	1.40	± 0.40	-	2.24	± 0.88	-
	-	-	-	**0.0001** ‡	-	-	**0.0001** ‡

Note: Bold values indicate statistical significance.

NRPS, Numerical Rating Pain Scale; SD, standard deviation; BCTQ, Boston Carpal Tunnel Syndrome Questionnaire; FSS, Functional Status Scale; ANOVA, analysis of variance.

† , Friedman test.

‡ , ANOVA repeated measures.

An ANOVA conducted on grip strength revealed a slight improvement in both groups, with a difference of 0.54 kg in the TG (*p* ˂ 0.001) and 0.64 kg (*p* = 0.01) in the CG (Table 2).

An ANOVA performed on the BCTQ-FSS in TG showed an improvement of 0.72 in function immediately after 10 treatment sessions and an overall improvement of 1.37 (*p* ˂ 0.001) at the end of treatment. In the CG, an improvement of 0.12 in function was also observed after 5 weeks and 0.37 (*p* = 0.01) immediately after treatment (Table 2).

The Wilcoxon test showed a statistically significant mean difference in pain intensity of 1.15 (95% confidence interval [CI]: 0.52–1.72; *p* = 0.001) between the treatment and CG after 5 weeks and 2 (95% CI: 1.47–2.55; *p* ˂ 0.001) after 10 weeks, respectively, whereas at baseline the difference was not significant 0.33 (*p* = 0.076), and the pain was more intense in the TG (Table 3).

**TABLE 3:** Group means and standard deviation for pain intensity, grip strength and functional status; between-group comparison; and effect of therapy.

Variable	Time of observation	Treatment group		Control group		*P*
		Mean	± SD	Mean	± SD	
Pain intensity (NRPS)	Baseline	5.17	± 1.71	4.83	± 1.95	0.076†
	5 weeks	3	± 1.76	4.15	± 1.87	**0.001** †
	10 weeks	1.52	± 1.50	3.52	± 1.76	**0.0001** †
Grip strength (Kg)	Baseline	20.29	± 3.71	19.59	± 3.08	**0.026** ‡
	5 weeks	20.50	± 3.71	19.91	± 3.40	**0.050** ‡
	10 weeks	20.84	± 3.56	20.23	± 3.29	**0.028** ‡
BCTQ-FSS	Baseline	2.77	± 0.88	2.62	± 0.88	**0.003** ‡
	5 weeks	2.05	± 0.76	2.50	± 0.86	**0.0001** ‡
	10 weeks	1.40	± 0.40	2.24	± 0.88	**0.003** ‡

Note: Bold values indicate statistical significance.

NRPS, Numerical Rating Pain Scale; SD, standard deviation; BCTQ, Boston Carpal Tunnel Syndrome Questionnaire; FSS, Functional Status Scale.

† , Wilcoxon test.

‡ , Paired *t*-test.

For grip strength, the paired *t*-test showed a statistically significant mean difference at baseline of 0.7 kg (95% CI: 0.087–1.30; *p* = 0.02), 0.59 kg (95% CI: 0.0007–1.17; *p* = 0.05) after 5 weeks and 0.60 kg (CI: 0.006–1.13; *p* = 0.02) after 10 weeks (Table 3).

For the BCTQ-FSS, the paired *t*-test showed a statistically significant mean difference between the two groups with a 0.15 (95% CI: 0.07–0.23; *p* = 0.003) at baseline, a 0.45 (95% CI: 0.31–0.59; *p* ˂ 0.001) after 5 weeks and a 0.84 (95% CI: 0.41–1.28; *p* = 0.003) difference at 10 weeks (Table 3).

## Discussion

The results of our randomised clinical trial are, for the most part, encouraging in terms of pain intensity and, to a lesser extent, functional status and grip strength. Indeed, both groups showed a significant improvement, but it was more pronounced in the TG. Over time, the reduction in pain intensity was considerably more improved immediately after treatment than at mid-treatment, with a significant mean difference of 3.65. In addition, both groups had a slight but still significant improvement in grip strength over time, which was more noticeable in the CG. Both groups showed a significant but more notable improvement in the TG than in the CG regarding functional status. For example, the function was improved by 1.37 on the BCTQ-FSS in the TG versus 0.12 on the CG. Furthermore, the therapy effectiveness between the groups was significant for all parameters in the different measurement times, except for pain intensity at baseline. These findings support our hypothesis that NMTs targeting the median nerve are more effective than joint mobilisation of the elbow not directed at the median nerve, especially in improving pain intensity and functional status when added to interfacing treatment.

The need for practical and conservative approaches to treating CTS has led several researchers to conduct a series of controlled trials and systematic reviews. These studies differ in terms of the therapy used in the CG and the parameters used to measure the treatment effectiveness. However, the findings have been inconsistent regarding the value of using NMTs for patients with moderate or mild CTS. Therefore, our randomised clinical trial aimed to assess the effectiveness of the NMTs versus joint mobilisation of the elbow not directed at the median nerve in women with bilateral moderate or mild CTS. The parameters investigated will now be discussed and compared with other studies.

We compared the therapy effect between the two groups based on paired samples. To our knowledge, this is the first time that such a sampling method has been used in similar studies, which could give a substantial statistical power gain to our study results (Stevens et al. 2018). Previous studies that tried to compare NMTs versus placebo effects used unpaired samples.

Our study results suggest that NMTs combined with carpal bone mobilisation have better effects on pain intensity, functional status and, to a lesser degree, grip strength than the placebo joint mobilisation technique not directed at the median nerve. These findings could be explained by NMTs’ desensitisation effects in decreasing pain levels and improving functional status (De-Las-Peñas et al. 2017). Furthermore, our sample was active with many tasks, whether at home (58.1%) or in a physical activity demanding occupation (19.4%). It is also possible that pain intensity reduction would have allowed patients to return earlier to their occupations, improving their grip strength and functional status. Mechanical interfacing treatment based on carpal bone mobilisation used in both groups could have interfered with NMTs and placebo mobilisation effects in explaining our results. Tal-Akabi and Rushton (2000) found that carpal bone mobilisation was statically significant in improving pain intensity in people with CTS but not functional status. Shem, Wong and Dirlikov (2020) showed that self-median nerve interfacing treatment improved 5 of 12 measures. This could be explained by the ability of carpal bone mobilisation to induce significant fluid dispersion, as Butler (1991) suggested, especially when added to NMTs (Boudier-Revéret et al. 2017; Schmid et al. 2012).

The positive effect on pain intensity, grip strength and functional status in the CG in our trial could be explained by the capacity of this mobilisation, which did not primarily target the median nerve, to blind the patients and boost their expectations of the treatment outcomes (Beauregard 2007; Benedetti 2006). This ability to blind participants is fundamental for the validity of placebo treatment in manual therapy (Hawk et al. 2002, 2005; Vernon et al. 2005). Bialosky et al. (2009) and Wolny et al. (2018) used placebo mobilisation in their CGs to quantify its effect versus NMTs. Bialosky et al. (2009) concluded that their placebo mobilisation successfully blinded the participants, which explains their results regarding functional status, grip strength and pain intensity in the CG. They also suggest that participants’ involvement in the study could explain these positive results. The CG in Wolny et al. (2018) did not improve in any parameter. Therefore, they mentioned no positive placebo effect, no increase in patients’ expectations and no success in blinding in their study. Patients who believe they are receiving active therapy may increase their expectations. However, it may generate less important results than the studied intervention, although sometimes significant, as in Bialosky et al. (2009) and our study’s case.

In some systematic reviews (Ballestero-Pérez et al. 2017; McKeon & Yancosek 2008; Núñez De Arenas-Arroyo et al. 2021) and earlier studies (Akalin et al. 2002; Brininger et al. 2007; Horng et al. 2011; Tal-Akabi & Rushton 2000; Wolny et al. 2017), NMTs did not decisively demonstrate their effectiveness in the conservative management of patients with mild or moderate CTS. Other studies found beneficial therapeutic results (Hamzeh et al. 2021; Wolny & Linek 2018; Wolny et al. 2017). This could be explained by the methodological discrepancies related to the type of NMTs used, the addition of different treatment modalities, therapy duration and whether home exercises were added to the programme. This methodological diversity makes it difficult to reach definitive conclusions in some systematic reviews.

Similar to our results, Wolny et al. (2017) compared the efficacy of manual therapy, including neurodynamic techniques of the median nerve, with electrophysical modalities (ultrasound and laser). The study results showed a significant improvement in pain intensity and functional status over time, corroborating our results. They found that pain decreased by 4.24 in the TG from pretreatment to post-treatment. This is similar to our findings of a 3.65 reduction between the baseline and the completion of therapy. Over time, both studies showed a BCTQ-FSS improvement that was nearly equivalent. Similar to what we demonstrated, Wolny et al. (2018) compared NMTs to a sham mobilisation in treating CTS and concluded the superiority of NMTs. In a similar study, Bialosky et al. (2009) showed that both NMTs and sham therapy groups gradually improved their functional status and pain intensity. As discussed previously, they attributed these results in their CG to the placebo mobilisation ability to increase patients’ expectations. De-la-Llave-Rincon et al. (2012) report a decrease in pain intensity following treatment based on, among other modalities, neurodynamic sliding mobilisation of the median nerve, which was used for the first 15 sessions in our trial for the TG. Recently, Hamzeh et al. (2021) evaluated the long-term effect of NMTs compared to exercise therapy and found a significant improvement in functional status and pain intensity in the therapy group. Yildirim et al. (2018) evaluated the effectiveness of adding kinesiotaping techniques to nerve and tendon gliding techniques. As in our study, both groups significantly improved their functional status over time, while the CG’s grip strength did not. Sim et al. (2019) conducted a study to assess the efficacy of orthosis alone versus an orthosis, nerve and tendon gliding exercises, and ultrasound therapy combination in the conservative management of CTS. Both groups experienced a significant reduction in symptom severity and functional status, corroborating our findings. As shown by our results, Horng et al. (2011) found a significant impact on pain intensity.

In contrast to our study findings, Wolny et al. (2018) and Bialosky et al. (2009) found no significant difference in pain intensity or functional status between their treatment and CGs. Sim et al. (2019) showed no meaningful difference in functional status. There was also no statistically significant difference in grip strength between the two groups in the studies by Hamzeh et al. (2021) and Yildirim et al. (2018). Horng et al. (2011) found that NMTs did not impact patients’ functional status. With a different result from the previous studies and our own, Brininger et al. (2007) confirmed their hypothesis that the orthosis groups would improve over time, regardless of whether neurodynamic techniques were used. In terms of positive impact, Hamzeh et al.’s (2021) study concluded that using NMTs directed at the median nerve helped avoid surgery in people with CTS.

In conclusion, NMTs seem more effective than placebo joint mobilisation of the elbow not directed at the median nerve, especially on pain intensity and functional status in women with mild to moderate CTS combined with mechanical interface treatment based on carpal bone mobilisation. Although there was a statistically significant difference between the two groups, the clinical difference was insufficient to recommend NMTs for improving grip strength in women with mild to moderate CTS.

### Study limitations

There are some limitations to our study. Unintentionally, we only had a female sample, which does not allow us to generalise the results to men with mild to moderate CTS. In addition, a double-blind clinical trial could not be carried out. The physiotherapist was aware of the side to be treated; however, the assessor was blinded. Thus, we cannot compare the NMTs or placebo mobilisation effects to natural history. Lastly, as a long follow-up was not planned for this trial, no conclusions could be made regarding the long-lasting NMT effects.

## Conclusion

To reduce pain intensity and improve functional status in women with mild to moderate CTS, NMTs for the median nerve combined with carpal bone mobilisation seem to be more effective than placebo mobilisation not directed at the median nerve. However, both interventions were effective on all studied parameters. Further randomised controlled trials are necessary to reach a sufficiently large sample size to support our results.
